# *Enterococcus casseliflavus* KB1733 Isolated from a Traditional Japanese Pickle Induces Interferon-Lambda Production in Human Intestinal Epithelial Cells

**DOI:** 10.3390/microorganisms10040827

**Published:** 2022-04-15

**Authors:** Shohei Satomi, Daichi Kokubu, Takuro Inoue, Masaya Sugiyama, Masashi Mizokami, Shigenori Suzuki, Kazumoto Murata

**Affiliations:** 1 Department of Nature & Wellness Research, Innovation Division, KAGOME CO., LTD., 17 Nishitomiyama, Nasushiobara 329-2762, Tochigi, Japan; daichi_kokubu@kagome.co.jp (D.K.); takuro_inoue@kagome.co.jp (T.I.); shigenori_suzuki@kagome.co.jp (S.S.); 2 Genome Medical Science Project, National Center for Global Health and Medicine, 1-7-1 Kohnodai, Ichikawa 272-8516, Chiba, Japan; msugiyama@hospk.ncgm.go.jp (M.S.); mmizokami@hospk.ncgm.go.jp (M.M.); kmurata@jichi.ac.jp (K.M.); 3 Division of Virology, Department of Infection and Immunity, Jichi Medical University, 3311-1 Yakushiji, Shimotsuke 329-0498, Tochigi, Japan

**Keywords:** screening, pickles, *Enterococcus casseliflavus* KB1733, heat-killed bacteria, interferon-lambda

## Abstract

The association between lactic acid bacteria (LAB) and their immunostimulatory effects has attracted considerable attention; however, it remains unclear whether LAB can induce interferon-lambdas (IFN-λs) in human epithelial cells under conditions that do not mimic infection. In this study, we first employed a reporter assay to screen for a potential strain capable of inducing IFN-λ3 among 135 LAB strains derived from traditional Japanese pickles. Next, we assessed the strain’s ability to induce the expression of IFN-λ genes and interferon-stimulated genes (ISGs), and to produce IFN-λs. As a result, we screened and isolated *Enterococcus casseliflavus* KB1733 (KB1733) as a potential strain capable of inducing IFN-λ3 expression. Furthermore, we clarified that KB1733 induced the expression of IFN-λ genes and ISGs related to antiviral functions, and that KB1733 induced IFN-λ1 and -λ3 expression in a dose-dependent manner up to 10 μg/mL. In addition, KB1733 significantly increased IFN-λ1 production compared to *Enterococcus casseliflavus* JCM8723^T^, which belongs to the same genera and species as KB1733. In conclusion, we isolated a unique LAB strain from traditional Japanese pickles that is capable of stimulating IFN-λ production, although further study is needed to investigate how KB1733 protects against viruses in mice and humans.

## 1. Introduction

Lactic acid bacteria (LAB) have historically been important mainly because of their utility in fermentation and food preservation as well as their health benefits. According to the definition suggested by the Food and Agriculture Organization (FAO)/World Health Organization (WHO), health-promoting LAB are called probiotics and are considered non-pathogenic live microorganisms that confer health benefits to the host when administered in adequate amounts [1]. Most LAB employed as probiotics belong to the genera *Lactobacillus* and *Enterococcus* [2]; however, their features are known to differ according to the strain [3]. Many studies have focused on the immunostimulatory effects of probiotics [4,5,6], especially on innate immune responses, and some of them have been found to have anti-infectious effects in humans and animals [7,8]. Furthermore, heat-killed LAB were also reported to show immune-modulatory properties similar to those of probiotics [9,10], suggesting that these could be used more effectively than live LAB because of their pharmaceutical advantages in terms of transport and storage [11]. Therefore, isolating unique heat-killed LAB strains that can strengthen the immune system may contribute to the development of functional foods and drinks that people could utilize to prevent infectious diseases in their daily lives [12].

The type III interferon (IFN) family, including IFN-λ1, -λ2, -λ3 (also known as interleukin (IL)29, IL28A, and IL28B, respectively) and -λ4 [13], has recently attracted a lot of attention as a crucial factor in the innate immune system. One subunit of the heterodimeric receptor of IFN-λ, IFN-λ receptor 1 (IFNLR1), is strongly expressed in the epithelial cells of mice and humans [14,15,16], and IFN-λ has been reported to provide antiviral protection to anatomic barriers such as the respiratory, hepatic, and gastrointestinal mucosa [17,18]. In addition, IFN-λ was found to readily induce the expression of IFN-stimulated genes (ISGs) such as myxovirus resistance 1 (Mx 1) and oligoadenylate synthetase 1 (OAS1) in epithelial cells of most body sites [19,20]. Furthermore, a previous in vitro study indicated that IFN-λ exhibited a dose-dependent inhibitory effect on SARS-CoV-2 in Vero cells [21]. Another in vivo study showed that IFN-λ treatment prevented and cured persistent enteric murine norovirus infection [22]. Recently, Murata et al. found that the nucleotide analogs, hepatitis B virus (HBV) polymerase inhibitors, induced IFN-λ3 in the gastrointestinal (GI) tract [23], which showed anti-HBV effects [24]. Serum IFN-λ3 levels were reported to be increased in patients with acute hepatitis E virus (HEV), while the HEV itself induced IFN-λ3 in colon cancer cell lines [25].

These results suggest that inducing IFN-λ production in epithelial cells before viral invasion could contribute to infection prevention. Previous studies have investigated the effects of LAB on IFN-λ induction in human dendritic cells [26] or porcine epithelial cells under conditions that mimic infection [27]. However, few studies have focused on the effects of LAB that can induce IFN-λs in human epithelial cells without mimicking infection. The aim of this study was to isolate potent and unique LAB strains with the ability to induce IFN-λs and exert antiviral effects on human intestinal epithelial cells. To our knowledge, this is the first study to investigate the effect of heat-killed LAB on IFN-λ production in human intestinal epithelial cells.

## 2. Materials and Methods

### 2.1. To Screen for the LAB Strain Capable of Inducing the Gene Expression of IFN-λ3

#### 2.1.1. Strains, Growth Conditions, and Sample Preparation

A total of 135 LAB strains were evaluated during the screening (Supplementary Table S1). All LAB strains belonged to the Department of Nature and Wellness Research, Innovation Division, KAGOME CO., LTD. (Nasushiobara, Tochigi, Japan). Our strains were isolated from traditional Japanese pickles collected throughout Japan, stored at −80 °C, and freeze-dried until use [28]. The control strain, *Lactococcus lactis* subsp. *lactis* JCM5805^T^ [26], was provided by the RIKEN BRC through the National BioResource Project of the MEXT/AMED, Japan. Each bacterium was inoculated with 1% (*v*/*v*) of a thawing glycerol stock in 10 mL de Man, Rogosa, and Sharpe (MRS) broth (Difco, Detroit, MI, USA) and incubated for 20 h at 30 °C. Next, 4 mL of cell culture was inoculated in 36 mL MRS medium and incubated for 20 h at 30 °C. The cell cultures were washed with sterilized saline and water, followed by centrifugation at 4 °C and 2590× *g* for 10 min (himac22G, R10A2, Eppendorf Himac Technologies, Hitachinaka, Ibaraki, Japan). To prepare heat-killed bacteria, washed bacteria were autoclaved at 100 °C for 30 min (Iwaki, Tokyo, Japan) and freeze-dried. Each freeze-dried sample was diluted with Dulbecco’s modified Eagle’s medium (DMEM) (FUJIFILM Wako Pure Chemical Co., Ltd., Osaka, Japan) to a concentration of 10 μg/mL and used in the assay. Adefovir dipivoxil (ADV) (Funakoshi, Tokyo, Japan) was used as a positive control because it had previously been reported to induce IFN-λs in WiDr cells [23]. Dimethyl sulfoxide (DMSO, 0.1%) (Sigma-Aldrich, St. Louis, MO, USA) was used as a negative control.

#### 2.1.2. Cells

We used a WiDr-Luc-IL28B cell line produced by Murata, K. The WiDr cells, which are human colon epithelial cell lines, were transfected with pGL4.28 (luc2CP/minP/Hygro) carrying the IL-28B promoter region [29]. The WiDr-Luc-IL28B cells were maintained in D-MEM supplemented with 10% fetal bovine serum (Life Technologies, Grand Island, NY, USA) and 0.5% hygromycin B (FUJIFILM Wako Pure Chemical Co., Ltd., Osaka, Japan) at 37 °C in the 5% CO_2_ gassed incubator. WiDr cells were selected for the screening and the functional assays of IFN-λs since WiDr cells were the most potent cell lines to induce IFN-λs among the five human colon cancer cell lines we tested [23].

#### 2.1.3. Cell Culture with Samples and Reporter Assay for IFN-λ3

WiDr-Luc-IL28B cells were seeded at 5 × 10^5^ cells/well, with a total volume of 100 μL in a 96-well black plate, CulturPlate-96F (PerkinElmer, Inc., Waltham, MA, USA), and incubated at 37 °C in a 5% CO_2_ gassed incubator for 48 h. After removal of the culture supernatants, the prepared samples were added into each well (4 wells/sample) and co-cultured for another 48 h. Then, after removal of the culture supernatant, the DMEM medium was added to each well at a volume of 50 μL/well, followed by the Steady-Glo^®^ Luciferase Assay System (Promega, Madison, WI, USA) at a volume of 50 μL/well; the 96-well black plate was then left for 5 min at 21 °C. After 5 min, the plate was placed in a CentroXS3 LB960 microplate luminometer (Berthold Japan, Tokyo, Japan), and the luciferase activity in each well was measured.

### 2.2. To Assess the Ability of Enterococcus casseliflavus KB1733 to Produce IFN-λs and Induce Expressions of IFN-λ Genes and ISGs

#### 2.2.1. Strains, Growth Conditions, and Sample Preparation

KB1733 was selected for further analysis. The control strain, *Enterococcus casseliflavus* JCM8723^T^, was provided by the RIKEN BRC through the National BioResource Project of the MEXT/AMED, Japan. The growth conditions, sample preparation, and positive control were the same as those described above. For the production of IFN-λ1, -λ2, and -λ3, each freeze-dried sample was diluted with D-MEM at concentrations of 0, 1, 3, 10, and 30 μg/mL and applied to the assay. For the expression assay of IFN-λ genes and ISGs, each freeze-dried sample was diluted with D-MEM at a concentration of 10 μg/mL.

#### 2.2.2. Cells

WiDr cells [23] were maintained in D-MEM (FUJIFILM Wako Pure Chemical, Osaka, Japan) supplemented with 10% fetal bovine serum (Life Technologies, Grand Island, NY, USA) and 0.5% penicillin-streptomycin (Thermo Fisher Scientific, Waltham, MA, USA) at 37 °C in an incubator with 5% CO_2_.

#### 2.2.3. Cell Culture with Samples

The WiDr cells were seeded at 1 × 10^6^ cells/well, with a total volume of 2 mL in a 6-well plate (AGC Techno Glass, Tokyo, Japan), and were incubated at 37 °C in a 5% CO_2_ gassed incubator for 48 h. After removal of the culture supernatants, the prepared samples were added into each well (1 well/sample) and co-cultured for another 48 h. The supernatant of the medium co-cultured with samples was used for the assay of IFN-λ production. Cells on a 6-well plate were used for the expression assay of IFN-λ genes and ISGs.

#### 2.2.4. Assay for Production of IFN-λ1, -λ2, and -λ3

The levels of IFN-λ1 and -λ2 in the supernatant were quantified by enzyme-linked immunosorbent assay (ELISA) using commercial kits, according to the manufacturer’s instructions. The IL-29 and the IL-28A Human ELISA Kits (Thermo Fisher Scientific, Waltham, MA, USA) were used for the IFN-λ1 and IFN-λ2 measurements, respectively.

The level of IFN-λ3 in the supernatant was quantified using a chemiluminescence enzyme immunoassay (CLEIA). The assay was performed according to a previously described method [30].

#### 2.2.5. Assay for Expressions of IFN-λ Genes and ISGs

Real-time PCR (RT-qPCR) was performed to quantify the selected mRNAs in the WiDr cells. Total RNA was extracted from all samples using TRIzol^®^ reagent (Thermo Fisher Scientific, Waltham, MA, USA) following the manufacturer’s instructions. The cDNA template was synthesized from the extracted RNA using the PrimeScript^®^ RT Reagent Kit (TaKaRa Bio, Kusatsu, Shiga, Japan) following the manufacturer’s instructions. The thermal cycling conditions for the reverse transcription reaction were 15 min at 37 °C, followed by 5 s at 85 °C. 

mRNA expression of IFN-λs, ISGs, and glyceraldehyde-3-phosphate dehydrogenase (*GAPDH*) as a housekeeping gene was measured using real-time PCR performed on a 7500 Fast Real-time PCR system (Applied Biosystems, Foster City, CA, USA) with TB Green^®^ Premix Ex Taq II (TaKaRa Bio, Shiga, Japan). The PCR thermal cycling conditions were 30 s at 95 °C, followed by 40 cycles of PCR reaction at 95 °C for 5 s and 60 °C for 30 s. Relative gene expression was calculated as fold induction compared to GAPDH expression. The primer sequences used are listed in Table 1.

### 2.3. Statistical Analysis

All statistical analyses were performed using EZR (Saitama Medical Center, Jichi Medical University, Saitama, Japan) [31]. EZR is a modified version (version 2.5–1) of the R Commander, designed to add statistical functions frequently used in biostatistics. A dose or time-course assessment of KB1733 (*n* = 6) was performed using the Steel test, and the treatment group data were compared with the control group data. Strain (KB1733 and JCM8723^T^) specificity for IFN-λ1 production (*n* = 4) was analyzed using the Tukey–Kramer method. All values are expressed as mean ± standard error (SE) (*n* = 4–6). In all analyses, *p-*values < 0.05 were considered statistically significant.

## 3. Results

### 3.1. To Screen for the LAB Strain Capable of Inducing the Gene Expression of IFN-λ3

**Figure 1:**
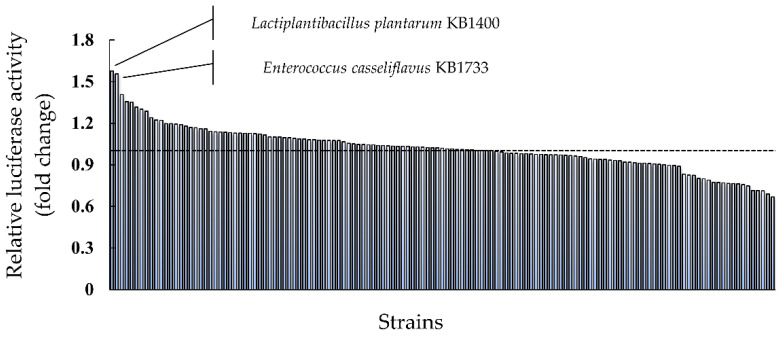
Screening for the potent LAB strain with an ability to induce the expression of the *IFN-λ3* gene among 135 strains. Dotted line indicates the value of relative luciferase activity of the control as 1.0. The value for each of the 135 strains is listed in Supplementary Table S2. Data from a single experiment are shown.

We screened for a potent LAB strain that could induce the expression of *IFN-λ3* using a reporter assay. Figure 1 and Supplementary Table S2 show the relative luciferase activity of each LAB strain (vs. JCM5805^T^). Among the 135 LAB strains evaluated in this study, two strains—*Enterococcus casseliflavus* KB1733 (KB1733) and *Lactiplantibacillus plantarum* KB1400—showed an approximately 1.5-fold higher induction of the IFN-λ3 gene expression as compared to that of JCM5805^T^. We performed the same experiment focusing on these two strains and found that only KB1733 induced luciferase activity at the same level as the first screening result (Supplementary Table S2).

### 3.2. To Assess the Ability of Enterococcus casseliflavus KB1733 to Induce IFN-λ Production and Expressions of IFN-λ Genes and ISGs

#### 3.2.1. Gene Expressions of IFN-λs

**Figure 2:**
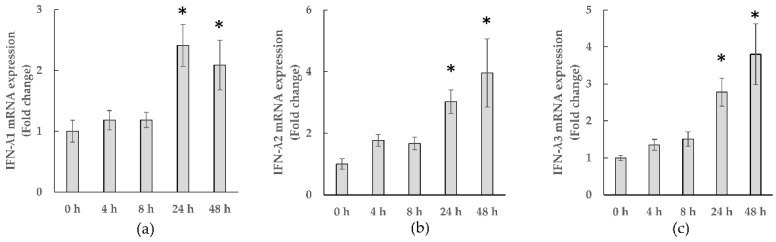
Gene expressions of (**a**) *IFN-λ1*, (**b**) *IFN-λ2*, and (**c**) *IFN-λ3*. The values are shown as means ± SE. Asterisks indicate a significant difference when compared to 0 h (*p*-value < 0.05). Data from six independent experiments are shown.

Figure 2 shows the time-course of IFN-λ gene expression in WiDr cells co-cultured with KB1733. KB1733 significantly increased the expression of all three genes (*IFN-λ1*, *-λ2*, and *-λ3*) at 24 h and 48 h compared to that at 0 h.

#### 3.2.2. Production of IFN-λ1, -λ2, and -λ3

**Figure 3:**
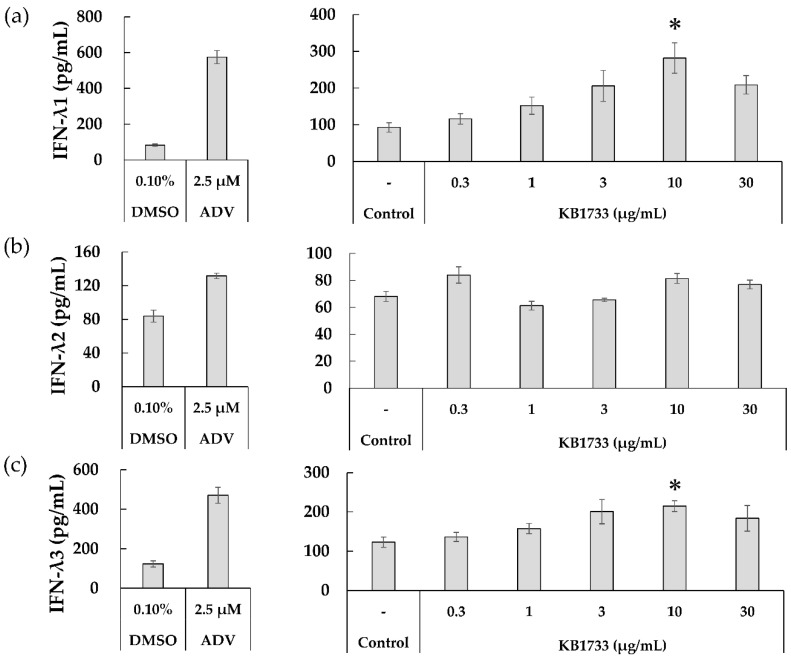
Production of (**a**) IFN-λ1, (**b**) IFN-λ2, and (**c**) IFN-λ3 after 48 h of KB1733 treatment. DMSO: Dimethyl sulfoxide employed as a negative control, ADV: Adefovir dipivoxil employed as a positive control. The values are shown as mean ± SE. Asterisks represent a significant difference when compared to control (DMEM) (*p*-value < 0.05). Data from six independent experiments are shown.

After checking the gene expression of IFN-λs in the WiDr cells co-cultured with KB1733, we assessed the dose-dependent effects of KB1733 on the production of IFN-λ1 and -λ2 using ELISA and of IFN-λ3 using CLEIA. Figure 3 shows the production of IFN-λ1, -λ2, and -λ3 in the cell culture supernatant after 48 h of KB1733 treatment. First, we confirmed that the assay worked by employing ADV and DMSO as positive and negative controls, respectively. KB1733 significantly induced both IFN-λ1 and λ3 at a concentration of 10 μg/mL (Figure 3a,c), although it did not significantly induce IFN-λ2 (Figure 3b).

#### 3.2.3. Expressions of ISGs

**Figure 4:**
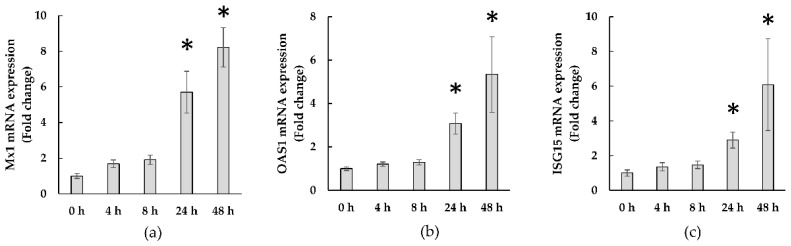
Gene expressions of (**a**) *Mx1,* (**b**) *OAS1*, and (**c**) *ISG15*. The values are shown as means ± SE. Asterisks mean a significant difference when compared to 0 h (*p*-value < 0.05). Data from six independent experiments are shown.

Figure 4 shows the time-course of ISG expression in WiDr cells co-cultured with KB1733. KB1733 significantly increased the expression of all three genes (*Mx 1*, *OAS1*, and *ISG15*) at 24 h and 48 h compared to that at 0 h.

#### 3.2.4. Strain Specificity for IFN-λ1 Production

To identify the strain specificity for IFN-λ production, we assessed the difference in IFN-λ1 production between KB1733 and JCM8723^T^.

**Figure 5:**
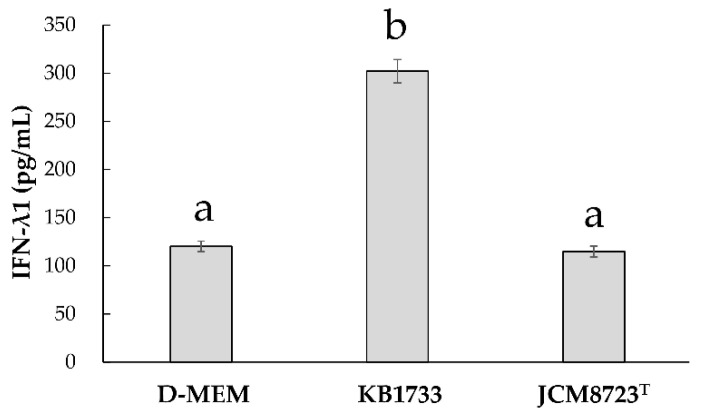
Strain specificity for IFN-λ1 production after 48 h of KB1733 or JCM8723^T^ addition (*n* = 4). The values are shown as mean ± SE. The treatments are significantly different if they do not share the same letter (**a**,**b**) (*p-*value < 0.05). Data from four independent experiments are shown.

Figure 5 shows IFN-λ1 production by the WiDr cell co-cultured with KB1733 or JCM8723^T^. IFN-λ1 production with D-MEM, KB1733, and JCM8723^T^ were 120.3 ± 10.9, 302.0 ± 24.4, and 114.7 ± 11.4 pg/mL, respectively. KB1733 significantly increased IFN-λ1 production compared to D-MEM or JCM8723^T^. However, JCM8723^T^ did not significantly increase IFN-λ1 production compared to D-MEM.

## 4. Discussion

The association between LAB and its immunostimulatory effects has attracted considerable attention; however, there are few studies analyzing whether LAB can induce IFN-λ expression in human epithelial cells without mimicking infection. In the present study, we aimed to identify heat-killed LAB with the potential ability to induce IFN-λ and antiviral-related genes in WiDr cell lines without stimulation by mimicking viral RNAs. We selected KB1733, which exhibited the strongest ability to induce *IFN-λ3* gene expression among a total of 135 strains isolated from traditional Japanese pickles. We further clarified that KB1733 could induce IFN-λ1 and -λ3 production in a dose-dependent manner up to 10 μg/mL. In addition, KB1733 induced the expression of genes such as IFN-λs and ISGs related to antiviral functions.

Fermented foods such as pickles and miso (fermented soy) are common in Japanese cuisine and have been recognized as safe for consumption. Pickles are foods fermented with LAB [32]. Fermented vegetables such as pickles are therefore potential sources of beneficial LAB. KB1733 was derived from a pickle, *wasabina-duke,* made from *Brassica juncea*. Although 10 other strains evaluated in this study were derived from the same source of isolation as KB1733, they did not enhance luciferase activity as high as this strain. Therefore, the IFN-λ-stimulating effects of LAB seem to depend on the strain itself rather than the source of isolation. In addition, we investigated the difference in IFN-λ1 production between KB1733 and JCM8723^T^ and found that IFN-λ1 production induced by KB1733 was significantly higher than that induced by JCM8723^T^. This means that the IFN-λ1-inducing effect did not depend on the genera and species but on strain specificity. The results of this study prove that we were able to successfully isolate a unique LAB strain from traditional Japanese pickles to stimulate IFN-λ production.

This study showed that KB1733 induced the production of IFN-λ1 and -λ3 in a dose-dependent manner up to 10 μg/mL, but this dose-dependency was not observed at a higher dose. The underlying mechanisms remain unclear, but a plausible explanation could be that a higher dose of KB1733 could impair IFN-λ production. This phenomenon is consistent with a previous study’s findings that *Lactiplantibacillus pentosus* S-PT84 could induce interleukin-12 in a dose-dependent manner, but not at a higher dose [33]. This issue requires further exploration in the future.

Although KB1733 enhanced the expression of IFN-λs, it did not significantly induce IFN-λ2 protein. A previous study comparing the biological activity of different IFN-λ subtypes clearly showed that IFN-λ3 was equal to or more potent than IFN-λ1 with respect to antiviral activity [34]. This study also showed that the specific activity of IFN-λ3 was 16-fold higher than that of IFN-λ2 despite a 96% sequence similarity between IFN-λ2 and -λ3 [34]. We considered that in terms of antiviral activity, KB1733 was a potent immunostimulant since it could promote the secretion of IFN-λ1 and -λ3 rather than IFN-λ2.

Furthermore, the expression of IFN-λ genes and ISGs was upregulated by KB1733. The results showed that the expression of all three ISGs (*Mx1, OAS1,* and *ISG15*) evaluated in this study at 24 h at the latest was significantly enhanced by KB1733. Moreover, our findings are consistent with a previous review describing IFN-λ-induced ISG expression [35]. Namely, KB1733 seems to induce ISGs, at least via IFN-λ induction. ISGs interfere with viral replication and provide immune defense to the host [23,35]. Considering these factors, KB1733 might be a possible candidate for enhancing host immunity against viral infections. In fact, Indo et al. showed that pre-treatment of porcine epithelial cells with *Legilactobacillus salivarius* strains before a rotavirus challenge could enhance the gene expression of *IFN-λ3*, *Mx 1*, and *OAS1* and significantly reduced both the rotavirus titer and infection rate [27]. In addition, Murata et al. investigated the inducing sites of IFN-λ3 using several cell lines that originated from different organs such as colon, lungs, skin, stomach, liver, and lymphocytes; they concluded that orally administered ADV induced IFN-λ3 in the GI tracts, which in turn induced ISGs in hepatocytes [23] although the precise mechanism remains unclear. When this phenomenon is extrapolated to KB1733, the putative mechanism of action for KB1733 application is presumably as follows: orally administered KB1733 reaches intestinal epithelial cells and induces IFN-λ production there. Subsequently, IFN-λs induced by KB1733 could, directly or indirectly through ISG, provide protection to the host against viral infections in the GI tract as well as other organs such as the liver or the lungs, to which IFN-λs are carried via blood flow. We intend to further explore how and whether KB1733 exerts protective effects against viral infection.

Nonetheless, it is unclear how KB1733 induces IFN-λ production and/or expression. In this study, we used the heat-killed and freeze-dried powder of KB1733 to investigate its IFN-λ-inducing effects. The powder likely includes DNA, RNA, and peptidoglycan derived from KB1733. Previous research has shown that nucleic acids derived from *E. faecalis* EC-12 induce IL-12 production in a murine macrophage cell line [36]. Another study showed that peptide glycans purified from *Lactobacillus salivarius* Ls33 induced IL-10 production in mice [37]. As described above, some studies have indicated a relationship between LAB components and their immunomodulatory properties. In this study, we did not clarify how KB1733 induced IFN-λ1 and -λ3 production. However, based on the fact that nucleotide analogs [23] or HEV [25] induce IFN-λ3 in the GI tract, it is intriguing to consider that host cells can recognize KB1733 as viral mimetic.

This study had several limitations. First, the application of KB1733 in the food industry requires several safety assessments. The genus Enterococci is assigned to risk group 2, which contains microorganisms that carry virulence factors [38]. Therefore, they are considered to be reservoirs for the dissemination of antibiotic resistance and pathogenic genes throughout the food chain [39]. In the future, we should perform a whole genome analysis of KB1733 to clarify whether this strain harbors antibiotic and virulence genes. Second, we only performed a screening for 133 strains once. Therefore, the possibility that we had lost certain hit strains inducing the gene expression of IFN-λ3 could not be eliminated; however, this screening test enabled us to quickly isolate *Enterococcous casseliflavus* KB1733 from our LAB libraries. Third, although we observed significant induction of IFN-λ genes by KB1733 in colon cancer cell lines as well as ISGs, which are the downstream genes of IFN-λs, we did not investigate whether these inductions of cytokines and ISGs are sufficient to elicit favorable responses against viral invasion. Therefore, further studies are required to translate the use of KB1733 to human applications aimed at improving human health.

## 5. Conclusions

Among the screened 135 isolates derived from traditional Japanese pickles, *Enterococcus casseliflavus* KB1733 exhibited the ability to induce IFN-λ and ISG gene expression. In this study, we isolated a unique LAB strain from traditional Japanese pickles capable of stimulating IFN-λ production, although further studies are needed to investigate how KB1733 can provide viral protection in mice and humans.

## Figures and Tables

**Table 1:** Primers sequences ^1^.

Genes	Primer Sequence (5′→3′)	
Human IL-29 *(IFN-λ1)*	F	GCCTCCTCACGCGAGACCTC
	R	GGAGTAGGGCTCAGCGCATA
Human IL-28A *(IFN-λ2)*	F	TCTGGAGGCCACCGCTGACA
	R	TGGGCTGAGGCTGGATACAG
Human IL-28B *(IFN-λ3)*	F	TGGCCCTGACGCTGAAGGTT
	R	CGTGGGCTGAGGCTGGATAC
Homo sapiens MX dynamin like GTPase 1 *(MX1)*	F	TACCAGACTCCGACACGAGTTCC
	R	GATTTGCTGTTTCACGATTGTCTCA
Homo sapiens 2′-5′-oligoadenylate synthetase 1 *(OAS1)*	F	AGAGCCTCATCCGCCTAGTCAA
	R	GCTCCCAAGCATAGACCGTCA
Interferon stimulated gene 15 *(ISG15)*	F	TGGACAAATGCGACGAACCTC
	R	CTGCGGCCCTTGTTATTCCTC
Glyceraldehyde 3-phosphate dehydrogenase *(GAPDH)*	F	TGGTGAAGACGCCAGTGGA
	R	GCACCGTCAAGGCTGAGAAC

^1^ F: Forward, R: Reverse.

## Data Availability

The data presented in this study as well as the bacterial strains used are available upon request from the corresponding author.

## Supplementary Materials

The following are available online at https://www.mdpi.com/article/10.3390/microorganisms10040827/s1. Table S1: Sources of isolation, genera, species, and names of the 135 strains evaluated in this study. Table S2: Relative luciferase activities of the 135 LAB strains.
