# Supplementary material for: Enterococcus casseliflavus KB1733 Isolated from a Traditional Japanese Pickle Induces Interferon-Lambda Production in Human Intestinal Epithelial Cells
Source: Microorganisms. 2022 Apr 15;10(4):827. doi: 10.3390/microorganisms10040827 (PMC9029449; doi:10.3390/microorganisms10040827)
Supplement: Supplementary file 1 [file microorganisms-10-00827-s001.zip › microorganisms-1643468-supplementary.pdf]

Satomi S. *et al*., *Enterococcus casseliflavus* KB1733 isolated from a traditional Japanese pickle induces interferon-lambda production in human intestinal epithelial cells: Supplementary Files.

Supplementary Tables S1–S2.

Table S1. Sources of isolation, genera, species, and names of the 135 strains evaluated in this study[1].

No.	**Sources of isolation (Prefecture in Japan);** main materials	**Genera, species**	**Strain name**
1		*Lactiplantibacillus pentosus*	KB625
2		*Lactiplantibacillus paraplantarum*	KB642
3	**Shiba-zuke (Kyoto);** Eggplant (*Solanum melongena*), Japanese basil (*Perilla frutescens* var. *crispa*), and salt.	*Companilactobacillus alimentarius*	KB653
4		*Lactiplantibacillus plantarum*	KB661
5		*Levilactobacillus brevis*	KB670
6		*Pediococcus ethanolidurans*	KB696
7		*Levilactobacillus brevis*	KB699
8		*Leuconostoc mesenteroides*	KB712
9		*Latilactobacillus sakei*	KB732
10	**Nozanawa-zuke (Nagano);** Cruciferous vegetable (*Brassica rapa* var. *hakabura*) and salt.	*Latilactobacillus curvatus*	KB738
11		*Weissella koreensis*	KB746
12		*Latilactobacillus curvatus*	KB753
13		*Latilactobacillus sakei*	KB757
14		*Lactiplantibacillus plantarum*	KB759
15		*Weissella koreensis*	KB770
16		*Enterococcus mundtii*	KB772
17		*Carnobacterium maltaromaticum*	KB775
18		*Latilactobacillus curvatus*	KB778
19	**Seisai-zuke (Yamagata);** Crufiferous vegetable (*Brassica juncea* var. *integrifolia*) and salt.	*Weissella koreensis*	KB798
20		*Latilactobacillus sakei*	KB807
21		*Leuconostoc mesenteroides*	KB835
22		*Leuconostoc mesenteroides*	KB851
23		*Leuconostoc carnosum*	KB854
24		*Leuconostoc sp.*	KB862
25		*Loigolactobacillus coryniformis*	KB879
26		*Companilactobacillus alimentarius*	KB881
27	**Takana-zuke (Fukuoka);** Cruciferous vegetable (*Brassica juncea* var. *integrifolia*) and salt.	*Lactiplantibacillus pentosus*	KB888
28		*Levilactobacillus namurensis*	KB897
29		*Levilactobacillus brevis*	KB898
30		*Lactiplantibacillus plantarum*	KB899
31		*Pediococcus parvulus*	KB920
32		*Pediococcus ethanolidurans*	KB956
33		*Latilactobacillus curvatus*	KB977
34		*Latilactobacillus sakei*	KB989
35		*Limosilactobacillus fermentum*	KB990
36	**Akakabu-zuke (Gifu);** Turnip (*Brassica rapa* var. *glabra*), cucumber (*Cucumis sativus*), and salt.	*Leuconostoc mesenteroides*	KB1002
37		*Lactiplantibacillus plantarum*	KB1015
38		*Lactiplantibacillus paraplantarum*	KB1017
39		*Lentilactobacillus buchneri*	KB1018
40		*Levilactobacillus parabrevis /hammessi*	KB1024
41		*Pediococcus parvulus*	KB1025
42		*Levilactobacillus brevis*	KB1029

Table S1. Continued

No.	Sources of isolation (Prefecture in Japan); main materials	Species of LAB	Strain name
43	**Suguki (Kyoto);** Turnip (*Brassica rapa* var. *neosuguki*) and salt.	*Levilactobacillus brevis*	KB290
44		*Limosilactobacillus fermentum*	KB1036
45		*Pediococcus ethanolidurans*	KB1046
46		*Levilactobacillus namurensis*	KB1049
47		*Levilactobacillus brevis*	KB1066
48		*Companilactobacillus alimentarius*	KB1087
49		*Lentilactobacillus buchneri*	KB1088
50		*Companilactobacillus farciminis/crustorum*	KB1089
51		*Levilactobacillus parabrevis*	KB1120
52		*Lactiplantibacillus plantarum*	KB1125
53		*Levilactobacillus parabrevis/hamesii*	KB1133
54		*Loigolactobacillus coryniformis*	KB1137
55		*Lactiplantibacillus pentosus*	KB1148
56		*Pediococcus acidilactici*	KB1150
57		*Limosilactobacillus reuteri*	KB1153
58		*Lacticaseibacillus paracasei*	KB1161
59		*Companilactobacillus alimentarius*	KB1163
60		*Levilactobacillus parabrevis*	KB1164
61		*Lactiplantibacillus pentosus*	KB1167
62	**Asotakana-zuke (Kumamoto);** Cruciferous vegetable (*Brassica juncea* var. *integrifolia*) and salt.	*Pediococcus ethanolidurans*	KB1168
63		*Lactiplantibacillus plantarum*	KB1173
64		*Loigolactobacillus coryniformis*	KB1182
65		*Levilactobacillus brevis*	KB1183
66		*Levilactobacillus hammesii/parabrevis*	KB1191
67	**Pesora-zuke (Yamagata);** Eggplant, red pepper (*Capsicum annuum*), and salt.	*Latilactobacillus sakei*	KB1194
68		*Pediococcus parvulus*	KB1203
69		*Latilactobacillus curvatus*	KB1207
70		*Companilactobacillus nodensis*	KB1211
71		*Furfurilactobacillus rossiae*	KB1226
72		*Levilactobacillus brevis*	KB1218
73		*Lactiplantibacillus plantarum*	KB1239
74		*Lacticaseibacillus paracasei*	KB1240
75		*Loigolactobacillus coryniformis*	KB1242
76		*Latilactobacillus sakei*	KB1243
77		*Levilactobacillus namurensis*	KB1245
78		*Companilactobacillus nodensis*	KB1247
79		*Companilactobacillus paralimentarius*	KB1251
80		*Lactiplantibacillus plantarum*	KB1253
81		*Enterococcus avium*	KB1256
82		*Pediococcus ethanolidurans*	KB1268
83		*Paucilactobacillus suebicus*	KB1278
84	**Inekokina-zuke (Nagano)**; Inekokina (*Brassica rapa L.* var. *hakabura Kitam.*) and salt.	*Latilactobacillus curvatus*	KB1292
85		*Latilactobacillus sakei*	KB1300
86		*Leuconostoc mesenteroides*	KB1330

Table S1. Continued.

No.	**Sources of isolation (Prefecture in Japan);** main materials	**Species of LAB**	**Strain name**
87	**Shakushina-zuke (Saitama);**	*Leuconostoc mesenteroides*	KB1350
88	Shakushina (*Brassica rapa*) and salt.	*Latilactobacillus sakei*	KB1351
89		*Carnobacterium gallinarum*	KB1371
90		*Enterococcus mundtii*	KB1372
91		*Levilactobacillus brevis*	KB1389
92	**Kuki-zuke (Mie and Wakayama);**	*Enterococcus termitis*	KB1396
93	Taro stalk (*Colocasia esculenta*), Japanese basil, plum	*Lactiplantibacillus pentosus*	KB1397
94	(*Prunus mume*) and salt.	*Lactiplantibacillus plantarum*	KB1400
95		*Levilactobacillus brevis*	KB1420
96		*Limosilactobacillus fermentum*	KB1433
97		*Pediococcus ethanolidurans*	KB1434
98		*Levilactobacillus namurensis*	KB1436
99	**Nanohana-zuke (Shiga);**	*Levilactobacillus namurensis*	KB1469
100	Nanohana (*Brassica rapa*) and salt.	*Lactiplantibacillus plantarum*	KB1471
101		*Companilactobacillus alimentarius*	KB1481
102		*Ligilactobacillus acidipiscis*	KB1488
103		*Levilactobacillus hamessii/parabrevis*	KB1493
104		*Companilactobacillus versmoldensis*	KB1494
105	**Tsudakabu-zuke (Shimane);**	*Latilactobacillus sakei*	KB1495
106	Turnip (*Brassica rapa* var. *glabra*) and salt.	*Leuconostoc carnosum*	KB1518
107		*Lactiplantibacillus paraplantarum*	KB1524
108		*Lactiplantibacillus plantarum*	KB1540
109		*Leuconostoc lactis*	KB1545
110		*Leuconostoc mesenteroides*	KB1549
111		*Levilactobacillus brevis*	KB1552
112		*Leuconostoc citreum*	KB1564
113		*Latilactobacillus curvatus*	KB1589
114	**Hinona-zuke (Shiga);**	*Latilactobacillus curvatus*	KB1637
115	Hinona (*Brassica rapa*) and salt.	*Lactobacillus fuchuensis*	KB1649
116		*Leuconostoc mesenteroides*	KB1652
117		*Weissella hellenica/paramesenteroides*	KB1653
118		*Latilactobacillus sakei*	KB1662
119		*Levilactobacillus brevis*	KB1663
120		*Pediococcus parvulus*	KB1664
121		*Companilactobacillus alimentarius*	KB1670
122		*Loigolactobacillus coryniformis*	KB1686
123	**Otakabuna-zuke (Hiroshima);**	*Companilactobacillus alimentarius*	KB1692
124	Turnip leaf and salt.	*Loigolactobacillus coryniformis*	KB1701

Table S1. Continued.

No.	**Sources of isolation (Prefecture in Japan);** main materials	**Species of LAB**	**Strain name**
125	**Wasabina-zuke (Shizuoka);**	*Weissella paramesenteroides/hellenica*	KB1730
126	Wasabina (*Brassica juncea* var. *cernua*), sake lees, and	*Weissella paramesenteroides*	KB1731
127	salt.	*Enterococcus casseliflavus*	KB1733
128		*Lactiplantibacillus plantarum group*	KB1742
129		*Lacticaseibacillus paracasei*	KB1754
130		*Leuconostoc pseudomesenteroides*	KB1756
131		*Leuconostoc citreum*	KB1757
132		*Weissella hellenica/paramesenteroides*	KB1765
133		*Leuconostoc lactis*	KB1771
134		*Leuconostoc mesenteroides*	KB1773
135		*Lentilactobacillus hilgardii*	KB1781

[1]All LAB strains listed above have been maintained at the Research Institute, KAGOME CO., LTD. KB290 was deposited as strain *Levilactobacillus brevis* JCM17312 in the Japan Collection of Microorganisms.

Table S2. Relative luciferase activities of the 135 LAB strains[1].

Strain name	Relative luciferase activities (vs control)	Strain name	Relative luciferase activities (vs control)	Strain name	Relative luciferase activities (vs control)
KB1400	1.58	KB1518	1.08	KB1771	0.97
	1.25 (2nd measurement)	KB775	1.07	KB1087	0.97
KB1733	1.55	KB746	1.07	KB1765	0.96
	1.63 (2nd measurement)	KB1493	1.05	KB290	0.96
KB898	1.41	KB1436	1.05	KB1066	0.95
KB888	1.36	KB1664	1.05	KB625	0.94
KB1649	1.35	KB977	1.05	KB1253	0.94
KB1194	1.32	KB1730	1.04	KB854	0.94
KB1589	1.30	KB1148	1.04	KB899	0.94
KB1564	1.29	KB642	1.04	KB1549	0.93
KB1552	1.24	KB699	1.04	KB1773	0.93
KB1161	1.22	KB1488	1.04	KB1495	0.93
KB1163	1.22	KB1686	1.03	KB1268	0.92
KB807	1.20	KB1692	1.03	KB881	0.92
KB1164	1.20	KB956	1.03	KB1540	0.92
KB712	1.19	KB738	1.03	KB1481	0.91
KB851	1.19	KB1191	1.03	KB1088	0.91
KB1029	1.18	KB920	1.03	KB1781	0.91
KB879	1.17	KB1292	1.03	KB1434	0.91
KB1670	1.17	KB1433	1.02	KB1469	0.90
KB1167	1.16	KB759	1.02	KB1494	0.90
KB897	1.16	KB753	1.02	KB1397	0.90
KB1701	1.14	KB1663	1.02	KB1251	0.90
KB1652	1.14	KB1545	1.01	KB1242	0.89
KB1524	1.14	KB1168	1.01	KB696	0.83
KB989	1.13	KB670	1.01	KB1243	0.83
KB990	1.13	KB772	1.01	KB1372	0.82
KB1637	1.13	KB1420	1.01	KB1218	0.80
KB1207	1.13	KB1240	1.01	KB1226	0.80
KB1153	1.13	KB1089	1.00	KB1183	0.79
KB1150	1.13	KB1389	1.00	KB1350	0.77
KB1024	1.12	KB1046	1.00	KB1300	0.77
KB732	1.12	KB757	1.00	KB1245	0.77
KB1731	1.12	KB1247	1.00	KB1351	0.77
KB798	1.10	KB1256	0.99	KB1203	0.76
KB835	1.10	KB1036	0.99	KB1330	0.76
KB1133	1.10	KB1757	0.98	KB1278	0.76
KB1662	1.10	KB1371	0.98	KB1182	0.75
KB1017	1.10	KB1396	0.98	KB1239	0.72
KB778	1.09	KB1742	0.98	KB1173	0.72
KB1018	1.09	KB1211	0.98	KB1137	0.71
KB1002	1.09	KB1756	0.98	KB1125	0.69
KB862	1.08	KB1049	0.98	KB1025	0.67
KB1015	1.08	KB1471	0.97		
KB661	1.08	KB653	0.97		
KB770	1.08	KB1754	0.97		
KB1120	1.08	KB1653	0.97		

[1]Control strain; *Lactococcus lactis* subsp. *lactis* JCM5805^T.
